# Sir A. Carlisle's "Alledged Discovery" of the Use of the Spleen and Thyroid Gland

**Published:** 1830-10-01

**Authors:** 


### XXIII.

ALLEGED DISCOVERY OF THE USE OF THE SPLEEN AND OF THE THYROID GLAND. By Sir A. CARLISLE, F.R.S. President of the Royal College of Surgeons, &c. Octavo, stitched, pp. 23. Price Half-a-Crown. Title page, with red and blue lines alternately.

WHEN we see an individual with an emaciated body, and a pallid face studded with carbuncles, we always conclude that some of the vital organs are in a state of decay, or at all events, of *derangement*. The recollection of this observation flashed on our minds, when we contemplated a royal octavo title-page crossed with red and blue stripes on a white ground—like the tricoloured flag waving over the fading lily of the Bourbons!

Sir Anthony dedicates this notable "DISCOVERY" to the Earl of Egremont, as a respectful homage to a nobleman "who generously maintained through life Mr. André, a helpless man of genius of my profession." What Mr. André may have done, or left undone, thus to merit, the eleemosynary protection of an English nobleman, we know not;—but we confess that the present dedication, founded on such a basis, looks rather awful, especially when coupled with the following *splenetic* effusion.

"The nobility of England in former times were the munificent protectors of literature and science, and the same patronage is wanted in the passing age; because forward and rapacious writers now address their crude productions to an increasing multitude of superficial readers, leaving the more profound labours of studious men to a market glutted with frivolous books."—*Pref.*

That the literary market has been glutted with frivolous books, in all ages, is a remark as old as Homer, and as often repeated as the succession of the hours and days, since the blind bard's time. But that good books are suffered to fall into oblivion because the nobility of England have ceased to patronize them, we most positively deny. The case indeed is altered. The patronage of a great man cannot now, as formerly, protect stupidity and imbecility from the keen arrows of criticism, or raise fools and flatterers into butterfly distinction. Patronage, now-a-days, must be sought and gained (if gained at all) from the PUBLIC—and, although GENIUS must often wither in the shade of neglect, we hope its fostering angel will never wing her flight from the republic of letters to the aristocracy of wealth.

We entreat the reader's attention to the following passage in the preface of this half-crown harlequin.

"The talent for *scenting* discoveries before they become apparent to the *vulgar*, is not a worldly advantage; it excites the hatred of *ordinary* persons, and occasions the jealous opposition of rivals. I do not look for a favourable reception of my doctrines until they have been submitted to the *more competent* physiologists of the Continent, and on their disinterested judgment I confidently rely, not only for a due estimation of the explanation here given of the uses of the spleen and thyroid gland, but also for a just valuation of all the collateral illustrations which are here adduced."—*Pref.*

The vanity which breathes through the foregoing passage is truly laughable. The "*talent* for *scenting discoveries* before they become apparent to the VULGAR!!!" And then again, the ingratitude of the author's compatriots! He looks for no favourable reception among his own countrymen! No! It is only among foreigners that Sir Anthony expects a favourable reception. And why? We are ready to grant that riches, rank, and reputation, in the medical profession, are followed by envy and detraction, as regularly as the substance is followed by the shadow. Sir Anthony is a knight; and what is more, he has held the presidency of the College of Surgeons. But these dangerous eminences have been attained by men before Sir Anthony's "discovery" saw the light, and that without drawing upon them the jealousy, envy, and hatred of their countrymen. Mr. Abernethy was president of the College, and yet Englishmen respected his writings. Sir Astley Cooper attained the baronetage as well as the presidency, and still his writings were devoured by the profession, as soon as they issued from the press.* Sir Anthony has not shewn much tact or discrimination in the foregoing passage. He has, at one and the same time, insulted the *justice* of his countrymen, and the *judgment* of foreigners. If Englishmen neglect or repudiate the "alleged discoveries" of Sir Anthony, because he is a knight or a president, they are a despicable set of wretches:—if foreigners embrace them, when the whole body of the British medical profession treat them with contempt, they are fools.

And now let us glance at this wonderful "*alleged discovery*" of the functions of the spleen and thyroid gland. The "*discovery*" was read at the College of Physicians, and escaped our notice last year. It is an old friend, with scarcely a new face! Both Harvey and Stukely imagined that the principal office of the spleen was to furnish a focus of heat. M. Ribes, in an elaborate article on the spleen, in the great Dict. des Sciences Med. makes use of the following expressions, which we recommend to Sir Anthony's attention. "We are surprised at the mass of futilities sent forth respecting the use of the spleen. Are we to believe, with some writers, that it serves as counterbalance to the weight of the liver—that it serves, according to Cowper, to attenuate the blood—or, according to Harvey, to *heat* the same?"—Now the great "discovery" which the "*talent for scenting*" has enabled Sir Anthony to make is, neither more nor less than this—that the spleen is a kind of oven for heating the stomach and thereby assisting it in the concoction of our food! The thyroid gland, like the spleen, is a warmer or comforter, in its way. It communicates heat to the cold and bloodless cartilages of the larynx, and thereby improves the voice.

"The extreme supplies of red blood to the spleen, and its necessary high temperature, when compared with the scantiness of red blood allotted to the stomach and to the intestines, has occasioned a generally admitted inference, that the spleen transmits heat to the stomach above the rate of its own capacity to furnish heat, and hence it has been concluded by many persons that the heat derived to the stomach from the spleen is conducive to digestion."

We think it is rather unfavourable to the worthy knight's theory that, when the stomach is empty, the spleen is fullest of blood, and consequently is best calculated for its calorific function. On the other hand, when the stomach is full of food, the spleen is most exsanguious!

Sir Anthony is not content with giving us his discovery of the splenic and thyroid functions for half a crown—he throws half a dozen of other discoveries into the bargain. The hair, which "has been childishly deemed a mere ornament," is proved by our author to be a preventive of coup de soleil and cold. "Perhaps also the axillary tuft of hair is intended as a defence against cold to that *most exposed plexus of nerves* which supplies the arm." This is excellent! It evinces Sir Anthony's surprising "talent for *scenting* discoveries," even in the arm-pits of his own species!

We perceive that the worthy knight, while extremely liberal of his harsh insinuations against "ordinary persons," "jealous rivals," &c. in his own profession, takes especial care to praise himself to the skies. The opening address to the president of the College of Physicians is admirable.

"As the temporary officer of a kindred institution, I feel much satisfaction in making this early attempt to associate the endeavours of scholars and gentle-

---

* This we know to our cost. The worthy baronet, instead of *buying* any favourable notice from us by a present of his late work, left us to search about and buy it at the expense of two guineas and a half! It is almost the only modern work which we have been obliged to purchase—and yet we shall not abuse the Baronet for the contribution which he has thus levied on our pockets.—*Editor.*

men to promote the science of organic physiology. On addressing your highly distinguished body upon subjects which have interested medical philosophers during more than 2000 years, some introductory apology may be deemed proper, and I accordingly submit that my pretensions to this honor rest upon zealous studies and practical researches in comparative anatomy during youth, and upon unceasing physiological meditations through a long professional life."

So then this long life of "zealous studies," "practical researches," and "physiological meditations," has brought out the wonderful discovery that the spleen is an oven for heating the stomach!

Parturiunt montes---nascitur ridiculus mus.

